# Inadequate sleep as a contributor to type 2 diabetes in children and adolescents

**DOI:** 10.1038/nutd.2017.19

**Published:** 2017-05-08

**Authors:** C Dutil, J-P Chaput

**Affiliations:** 1 Healthy Active Living and Obesity Research Group, Children’s Hospital of Eastern Ontario Research Institute, Ottawa, Ontario, Canada

## Abstract

Lack of sleep is a modifiable risk factor for adverse health in humans. Short sleep duration and poor sleep quality are common in the pediatric population; the largest decline in sleep duration over the past decades has been seen in children and adolescents. The objective of the present narrative review was to provide for the first time an overview of the literature on sleep and its association with type 2 diabetes mellitus (T2D) biomarkers in children and adolescents. For this narrative review, 23 studies were retained (21 observational and 2 experimental studies). Notwithstanding the conflicting results found in these studies and despite being attenuated by adiposity level, maturity, sex and age, there is still some compelling evidence for an association between sleep duration (for both objective or subjective measurements of duration) and architecture with one or more T2D biomarkers in children and adolescents. The majority of the studies reviewed did focus on sleep duration and one or more T2D biomarkers in children and adolescents, but sleep architecture, more precisely the suppression of slow wave sleep and rapid eye movement sleep, has also been shown to be associated with insulin resistance. Only two studies looked at sleep quality, and the association between sleep quality and insulin resistance was not independent of level of adiposity. Future experimental studies will help to better understand the mechanisms linking insufficient sleep with T2D. Work also needs to be carried out on finding novel and effective strategies aimed at improving sleep hygiene and health outcomes of children and adolescents.

## Introduction

Sleep is an important component of physical and mental health in humans. However, sleep deprivation has become common in modern societies.^1, 2^ Children and adolescents sleep less now compared with decades ago, and the steeper decline in sleep duration is found in adolescents and on school days.^3^ Lack of sleep is associated with a wide range of adverse health outcomes, including obesity, cardiovascular disease, depression, poor academic achievement and reduced quality of life/well-being.^4, 5^ Healthy sleep comprises many dimensions, including adequate duration, good quality, appropriate timing and regularity and the absence of disturbances or disorders.^6^ Fortunately, insufficient sleep is a potentially remediable health risk. A better understanding of the connection between insufficient sleep and health across the life cycle is important to help inform the development of interventions aimed at improving sleep habits and, subsequently, health outcomes.

Type 2 diabetes mellitus (T2D) is a global public health concern and lifestyle changes, such as improving diet composition, increasing physical activity and weight management have traditionally been the cornerstones of prevention efforts. Owing to the lack of effectiveness of most programs, other modifiable determinants of the disease should be targeted for providing more comprehensive and tailored prevention strategies and hopefully optimize success. Healthy sleep is gaining recognition worldwide as an important lifestyle habit associated with the prevention of chronic diseases, including T2D.^7, 8, 9^ For example, a recent systematic review and meta-analysis showed that the risk of developing T2D associated with insufficient sleep was comparable to that of traditional risk factors, such as excess weight, family history of diabetes and physical inactivity.^10^ Thus there is increased recognition that sleep should be considered in clinical guidelines for T2D.

Several meta-analyses have confirmed the independent association between sleep duration and the risk of developing T2D in adults.^11, 12, 13^ A U-shaped dose–response relationship is observed between sleep duration and the risk of T2D in adults, with the lowest risk observed at a sleep duration of 7–8 h per day.^11, 12, 13^ The mechanisms that underlie these associations are not fully understood; however, insufficient sleep is associated with insulin resistance, increased food intake and impaired glucose tolerance.^14^ The mechanisms underlying the association between long sleep duration and increased T2D risk are more speculative and may be associated with other health problems that confound the relationship (for example, depression, undiagnosed medical disease and poor physical health).

Although many articles have previously reviewed the evidence linking sleep with T2D in adults, the present paper is the first to look at this association in children and adolescents. The pediatric population is particularly vulnerable to experiencing insufficient sleep and is the one that has seen the largest decline in sleep duration over the past decades.^1, 2, 3^ Furthermore, intervening at an early stage in life is better for preventing long-term health consequences and this paper provides insights into the scope of the problem in this population as well as possible benefits of interventions aimed at improving sleep on glucose homeostasis. Therefore, the objective of the present narrative review is to give an overview of the literature on sleep and its association with T2D in children and adolescents.

## Methods

We performed a literature search for publications using PubMed until 15 November 2016. Search strategies included the following key words: ‘children’, ‘adolescents’, ‘youth’, ‘sleep’, ‘glucose’, ‘insulin’, and ‘diabetes’. Only publications in English were considered. Reference lists of the relevant publications were cross-checked for additional publications. Studies were included if they examined the association between sleep (either duration, architecture and/or quality) and markers of glucose homeostasis (for example, insulin, glucose, C-peptide, homeostasis model assessment of insulin resistance (HOMA-IR), hemoglobin A1c (HbA1c), Matsuda index and so on). Studies with type 1 diabetes only were excluded. Studies that included children and adolescents up to 19 years of age for at least one exposure measurement point were retained. Studies that reported on clinical populations only were excluded (for example, patients with obstructive sleep apnea). Studies were included if they used either objective (for example, polysomnography (PSG), actigraphy) or subjective (for example, self-report, proxy report) measures of sleep duration and/or sleep quality. All study designs were considered with no sample size limitations. Published peer-reviewed original manuscripts and in-press manuscripts were eligible for inclusion. Gray literature (for example, book chapters, dissertations, conference abstracts) were excluded. Where multiple models were reported in the studies (for example, unadjusted and adjusted models), results from the most fully adjusted model are presented in the tables. A meta-analysis was planned if results were found to be sufficiently homogeneous in terms of statistical, clinical and methodological characteristics. However, it was determined that a meta-analysis was not possible because of high levels of heterogeneity for the above characteristics across studies, and a narrative synthesis is presented instead.

## Inadequate sleep as a contributor to type 2 diabetes in children and adolescents: observational evidence

The key characteristics and main findings of the 21 observational studies that were retained for this review are presented in Table 1. Among them, 13 studies showed negative associations between one or more sleep characteristic (for example, duration and/or architecture) and one or more T2D biomarkers, 10 studies used an objective sleep measurement (for example, PSG, actigraphy or Fitbit activity watch), 2 studies were longitudinal in design and 1 study explored the phenotypic link between sleep duration and insulin resistance. A little more than a third of the observational studies found no associations between one or two sleep characteristics and one or more T2D biomarkers.

### Sleep duration and glucose homeostasis

Ten studies out of the 21 reported no association between sleep duration and glucose homeostasis biomarkers^15, 16, 17, 18, 19, 20, 21, 22, 23, 24^ (Table 1). These findings appear to be independent of the method used to measure sleep duration (that is, subjective vs objective sleep duration measurement); six of these studies employed self- or parent-reported questionnaires,^16, 17, 18, 19, 20, 21^ two studies employed a combination of actigraphy and sleep log,^22, 23^ one study used PSG^15^ and one study provided its participants with a commercial health and activity watch (Fitbit), also accompanied with a sleep log.^24^ Although the type of measurement used to assess sleep duration may not be a critical limitation, there are other important factors to consider when examining the association between sleep and glucose homeostasis biomarkers in children and adolescents. These factors include maturation stage, physical activity, nutrition, screen time, other stressors and level of adiposity. Additionally, when measuring sleep duration, it may be useful to also measure sleep quality in order to evaluate if the number of hours are indeed insufficient or sufficient for each participant individually (one size does not fit all when we measure sleep duration; some individuals can cope better than others with less sleep than recommended). Sleep guidelines, discussed later in the present review, are presented as a range of hours for a particular age group; however, some children may require more or less sleep due to many factors (for example, genetics, environmental factors, health status and so on) than what the guidelines stipulate to feel rested. Thus, without assessing how tired each participant feels after a night of sleep, it is very hard to determine if the duration of sleep is indeed sufficient.

Despite the numerous studies with results showing no association between sleep duration and glucose homeostasis, both longitudinal studies demonstrated a significant association between short sleep duration and higher insulin resistance, independent of age and sex.^25, 26^ In the study by Cespedes *et al.*,^25^ the association between short sleep duration and higher insulin resistance disappeared after controlling for body mass index (BMI), suggesting that the association was moderated and partially explained by the children’s weight status. Conversely, in the longitudinal study by Hjorth *et al.*,^26^ the negative association between short sleep duration and insulin resistance remained after controlling for adiposity. Although the longitudinal results by Hjorth *et al.*^26^ were independent of adiposity level, their cross-sectional results were not. Given that only two longitudinal studies were published in the pediatric population on the association between sleep duration and glucose homeostasis indicators, with both showing conflicting evidence for the role of adiposity in this association, replication studies are needed to better understand if excess adiposity is an important mediator of this association or not. In cross-sectional studies, the role of adiposity in the relationship between sleep duration and insulin resistance is also ambiguous. It is of interest to note that out of the 11 cross-sectional studies^19, 26, 27, 28, 29, 30, 31, 32, 33, 34, 35^ that have reported an association between sleep duration and T2D biomarkers, only 4 studies^28, 32, 34, 35^ have presented results independent of adiposity and 2 studies^29, 31^ were conducted with obese children; however, in both studies short sleep duration was associated with insulin resistance independent of the level of adiposity. For instance, Flint *et al.*^29^ reported that short sleep duration (⩽6 h per night) in obese children was associated with higher peak insulin, higher fasting insulin and lower whole-body insulin sensitivity, independent of the level of obesity and age.

Javaheri *et al.*^30^ reported a U-shaped relationship between sleep duration determined by actigraphy and HOMA-IR; however, the findings were attenuated after controlling for waist circumference and only long sleep duration remained significantly associated with HOMA-IR. Similarly, Koren *et al.*^31^ also observed a U-shaped relationship in adolescents with obesity between sleep duration measured by PSG and three of the glucose homeostasis outcome measurements (that is, glycated hemoglobin, short-term hyperglycemia and long-term hyperglycemia). This relationship remained after controlling for obstructive sleep apnea syndrome and level of obesity. Moreover, Koren *et al.*^31^ found that a sleep duration between 7 and 8.5 h per night in obese children and adolescents was associated with optimal glucose homeostasis. Although these two studies^30, 31^ have reported a U-shaped relationship between sleep duration and T2D biomarkers, similar to the relationship seen in adults,^11, 12, 13^ other studies^19, 25, 26, 27, 28, 29, 32, 33, 34, 35^ in the pediatric population did not or could not report the linearity or the shape of the relationship between sleep duration and glucose homeostasis biomarkers.

A study by Tian *et al.*^34^ on the relationship between sleep duration measured by parent report and fasting glucose in children aged 3–6 years showed only an association when stratified by weight status after adjusting for numerous covariates, such as birth weight, gestational age, BMI, systolic blood pressure, parents’ education, parents’ BMI, breastfeeding at the age of 6 months, diet, diseases in the past 6 months and waist circumference. These findings are not surprising when considering that most studies, regardless of the methods or the study design used, have reported no association between sleep duration and blood glucose levels in children.^16, 17, 20, 22, 23, 24, 25, 28, 29, 30, 32, 36, 37^ Nevertheless, one study by Zhu *et al.*^35^ did find a negative association between glucose and sleep time after adjusting for adiposity, maturation, age, sex and obstructive sleep apnea. Overall, these studies indicate that glucose may not be the prime marker in the association between short sleep duration and T2D in the pediatric population.

A few studies in this section conveyed a different and noteworthy perspective on the relationship between sleep duration and T2D biomarkers. De Bernardi Rodrigues *et al.*^28^ used the hyperglycemic clamp technique on a subsample of adolescents to measure insulin sensitivity. Although they found no association between short sleep duration (<8 h per night) and insulin resistance in the overall sample, they observed a lower median IQR insulin sensitivity index in the subsample of adolescents with short sleep duration compared with those with ⩾8 h of sleep per night after controlling for age and sex.^28^ Also, Androutsos *et al.*^27^ reported increased HOMA-IR in children aged 9–13 years with a lifestyle characterized by short sleep duration (8.35±0.73 h per night), high screen time (⩾3.61±1.68 h per day) and high consumption of sugared-sweetened beverages (⩾222.96±222.81 g per day) after controlling for age, sex, Tanner stage, waist circumference, parental BMI and birth weight. It is, however, difficult to compare this study with the others in this section as they did not isolate the relationship between short sleep duration and insulin resistance.

Another study that stands alone in its findings is a study by Prats-Puig *et al.*^33^ who observed a significant negative association between sleep duration and HOMA-IR in children of a specific phenotype. The last observational study discussed in this section is by Matthews *et al.*^32^ who showed that both short sleep duration during weekdays and overall short sleep duration were associated with increased HOMA-IR after adjusting for sex, age, race, waist circumference *z*-scores and BMI residuals, while longer weekend sleep duration was not associated with HOMA-IR. This observational study reinforces the idea that ‘catch-up sleep’ on weekends is not sufficient to counteract the lack of sleep accrued during the week. They also investigated the association between sleep duration measured by actigraphy and sleep timing assessed by diary and insulin resistance/hyperglycemia in apparently healthy adolescents and observed a significant association between sleep fragmentation and higher glucose concentrations.^32^ Although this association in apparently healthy adolescents is, to our knowledge, novel, another study in obese adolescents with sleep breathing disorders^38^ and another one conducted in adolescents with circadian rhythm sleep disorders^39^ have also shown an association between sleep fragmentation and hyperglycemia. Matthews *et al.*^32^ also noted that the amount their adolescent participants slept each night was much less than the recommended 9 h. A plausible explanation, unexplored by the authors, is the well-documented shift toward evening chronotype that occurs during pubertal years, a shift that cannot solely be attributed to the 24/7 lifestyle but has a complex physiological basis that is evident in many mammals.^40, 41, 42^ This circadian shift occurring during adolescence, coupled with the imposed early rising to attend school, may be a factor to consider in the association between sleep duration and insulin resistance during pubertal years. However, there are no studies on this relationship in adolescents.

### Other sleep characteristics and glucose homeostasis

Additional sleep characteristics (for example, architecture and quality), other than duration, have been studied. Six studies^15, 18, 26, 29, 31, 35^ out of the 21 presented in Table 1 assessed sleep characteristics other than duration and examined their associations with glucose homeostasis in the pediatric population. In those studies, sleep quality was measured via questionnaire^18, 26^ and sleep architecture was measured by PSG.^15, 29, 31, 35^ Measurement tools to assess sleep characteristics are diverse and each tool has its own list of limitations and advantages that deserve careful consideration. For example, PSG is considered the gold-standard measurement for sleep characteristics and the diagnostic tool for obstructive sleep apnea syndrome in the pediatric population^43^; however, PSG studies have some limitations as they can be a poor representation of at-home sleep routine and PSG is an impractical measurement tool for prolonged measurement periods and epidemiological studies with large sample sizes.

Berentzen *et al.*^18^ showed no association between sleep quality (measured by questionnaire) and hemoglobin A1c in children aged 11–12 years. Of note, a substantial limitation of this study, disclosed by the authors, was the time gap between questionnaire completion and the blood sample (that is, up to a year), thereby possibly introducing errors that may include changes in sleep characteristics due to changes in season, in stressors and attainment of puberty, especially relevant to girls in this age group. However, it is impossible to categorically state if this time lapse between measurements had a substantial role in this study’s lack of association between sleep characteristics and hemoglobin A1c. The five other studies discussed in this section have found associations between sleep characteristics and T2D biomarkers.^15, 26, 29, 31, 35^ Sleep quality was also assessed by Hjorth *et al.*^26^ in their cross-sectional study showing a negative association between sleep quality and HOMA-IR, independent of several covariates but not adiposity. Sleep architecture was examined by Armitage *et al.*^15^ who showed that a decrease in the delta waves in non-rapid eye movement stage 1 (NREM1) were associated with increased insulin resistance, even after adjusting for BMI and Tanner stage. Likewise, a study by Zhu *et al.*^35^ in children and adolescents also found a negative association between higher NREM1and inefficient sleep and insulin sensitivity and glucose tolerance, after controlling for maturity and adiposity. Two other studies^27, 31^ also found sleep architecture to be associated with numerous T2D biomarkers. In adults, many factors, such as sex, age, ethnicity, sleep-disordered breathing, adiposity and smoking contribute to the variability in sleep architecture.^44^ Bearing in mind that no such studies on the heterogeneity of sleep architecture in children and adolescents have been carried out, the association between sleep architecture and insulin resistance in children and adolescents is similar to what is found in adults and deserves further investigation.

Overall, observational studies have provided valuable insights into the association between sleep and T2D in children and adolescents. Many studies in children and adolescents, discussed in this review, have reported a relationship between poor sleep duration and insulin resistance. Yet, the shape of the relationship remains unclear in the pediatric population. A more linear or J-shaped relationship rather than U-shaped may be plausible in this population and could be explained by the fact that kids are generally healthier and long sleep is often a marker of poor health in adults that may confound the associations reported. Sleep architecture, more precisely the suppression of slow wave sleep (SWS) and rapid eye movement (REM) sleep, has also been shown to be associated with insulin resistance. The role of adiposity in the relationship between sleep and T2D in the pediatric population still remains unclear. There is also evidence that plasma glucose is a secondary risk marker in the association between sleep and T2D in the pediatric population when compared with insulin resistance measurements. Given the mixed results, in the presented cross-sectional studies it would be advantageous to investigate the association between inadequate sleep and T2D biomarkers in children and adolescents in a prospective manner in order to better understand the chronic effect of sleep duration on insulin resistance in the pediatric population. Although, to our knowledge, no such studies exist in the pediatric population, Shan *et al.*^11^ recently reviewed the prospective evidence on sleep duration and T2D in adults. As a whole, the evidence reveals that the lowest risk of T2D in adults is associated with 7–8 h of sleep per night while both long and short sleep durations are associated with a greater risk of T2D.^11^

## Inadequate sleep as a contributor to T2D in children and adolescents: experimental evidence

Only two experimental studies have examined the effects of sleep restriction or disruption on glucose homeostasis in children and adolescents (Table 2). The crossover study by Klingenberg *et al.*^36^ demonstrated that three nights of sleep restriction (4 h per night) decreased insulin sensitivity compared with three nights of adequate sleep (9 h per night) in healthy male adolescents, despite having similar amount of SWS between the two conditions. However, the similar amount of SWS in both conditions came at the substantial loss of REM sleep duration in the sleep-restricted condition.^36^ In the other crossover study, Shaw *et al.*^37^ showed that SWS disruption did not affect fasting glucose, insulin or C-peptide levels and did not impair insulin sensitivity or beta-cell responsiveness at a mixed meal challenge in adolescents. In contrast with these results, a study conducted in young healthy weight adults revealed that after three nights of SWS suppression, insulin sensitivity decreased by nearly 25% from baseline.^45^ Additionally, in the study by Shaw *et al.*,^37^ the percentage of REM sleep was not statistically different between the two conditions (with vs without SWS disruption). Interestingly, a study in adults has shown that glucose utilization is higher during REM sleep than during SWS.^46^ Although there is insufficient evidence at present to draw a definitive conclusion based on only two experimental studies, it appears that REM sleep has an important role in glucose homeostasis in adolescents. Furthermore, healthy adolescents may be less prone to insulin resistance based on acute SWS disruption than young adults. However, disregarding SWS disruption as a potential T2D risk factor in the pediatric population may be hasty in a context of preventive medicine. Collectively, it appears evident that more experimental studies are needed in children and adolescents to better understand the effects of sleep restriction and fragmentation on T2D risk and the associated mechanisms.

## Mechanisms that may explain the association between inadequate sleep and T2D

**Figure 1:**
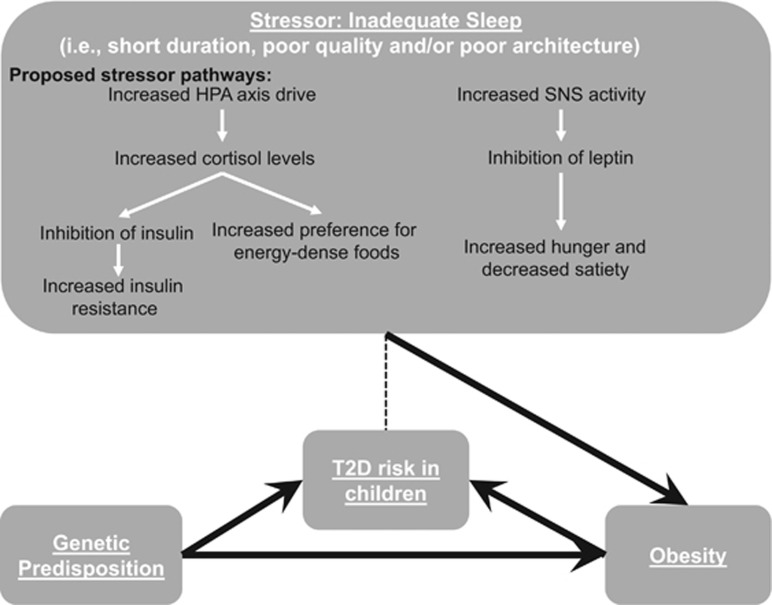
Proposed pathways that link inadequate sleep with T2D in the pediatric population. Note: The full arrows represent directional association between the components, while the dashed line refers to the possible association, based on the limited observational evidence, between inadequate sleep and T2D in children and adolescents. HPA, hypothalamic–pituitary–adrenal; SNS, sympathetic nervous system.

Studies in adults along with the contemporary emergence of evidence supporting an association between sleep deprivation and TD2 in children and adolescents has helped to partially elucidate the biological pathways. It is known that sleep in humans is a refractory period for the stress hormones cortisol, norepinephrine and epinephrine. The hypothalamic–pituitary–adrenal axis downregulates these stress hormones at night during sleep; however, if sleep is insufficient it results in higher cortisol levels during the day.^47^ Cortisol is involved in many metabolic processes, including inhibiting insulin production, thus increased levels of cortisol are associated with insulin resistance and an increased need for energy-dense foods.^14^ Although studies in adults have found an association between increased cortisol level in the evening and increased insulin resistance the following morning,^46, 48^ Klingenberg *et al.*^36^ found no differences in morning cortisol levels in healthy male adolescents following sleep restriction compared with the adequate sleep condition. In adults, an increased exposure to cortisol due to short sleep duration contributes to increased fat accumulation in the visceral region,^49^ which may be one reasonable explanation for the link between insufficient sleep, adiposity level and insulin resistance. Inadequate sleep is also thought to be a stressor for the autonomic nervous system by increasing the activity of the sympathetic nervous system. Although the hypothalamic–pituitary–adrenal axis downregulates stress hormones while we sleep, leptin (an anorexigenic hormone) is upregulated by adipocytes.^50^ However, when sleep is restricted, leptin similarly to insulin gets inhibited owing to the overdrive of the sympathetic nervous system in response to the stressor, namely, the lack of sleep. The inhibition of leptin leads to an increase in hunger and a decrease in satiety. These effects can lead to an increase in energy intake and weight gain over time. The pathway between insufficient sleep and T2D biomarkers through the variation of neuroendocrine and metabolic hormones is presented in Figure 1. Some other proposed mechanisms that link insufficient sleep to T2D biomarkers in adults include pro-inflammatory activity and increased ghrelin levels.^7, 13^

## Interventions aimed at improving sleep: possible benefits for improving glucose homeostasis?

Arora and Taheri^51^ recently reviewed the evidence on the efficacy of sleep improvement programs and their potential influence upon addressing obesity and metabolic disturbances. Collectively, the evidence in this field is scarce and much needs to be carried out on how best to improve sleep habits of children and adolescents, and whether such improvements translate into better glucose homeostasis outcomes. Of the 12 studies included in their review, 7 reported some positive sleep behavior changes postintervention.^51^ However, no studies to date have tried to determine if improving sleep leads to positive changes in glucose homeostasis in the pediatric population. This is certainly an important area to investigate in future studies.

In adults, Leproult *et al.*^52^ reported that 6 weeks of sleep extension improved insulin sensitivity in sleep-restricted adults. Whether similar effects can be observed in the pediatric population remains to be seen. The best approach to increase and/or improve sleep in the pediatric population is also unclear. However, recent findings are encouraging and demonstrate that intervening on sleep duration in the pediatric population is possible and can lead to improvements in appetite control and body weight regulation. For example, Hart *et al.*^53^ conducted a randomized crossover trial in 37 children aged 8–11 years and showed that, compared with decreasing sleep duration by 1.5 h per night, increasing sleep duration by 1.5 h per night over a week resulted in lower food intake and lower body weight. Tan *et al.*^54^ have also reported that a sleep hygiene education program was effective in improving sleep and decreasing BMI *z*-scores in children and adolescents aged 10–18 years with self-identified sleep problems. Future studies should go beyond energy balance and weight loss and determine whether such sleep intervention programs also improve metabolic function, such as insulin sensitivity.

Before embarking in large randomized controlled trials to test the efficacy of sleep improvement programs in the pediatric population, further pilot studies are needed. Programs that apply an evidence-based psychological theory of behavior change (for example, cognitive behavioral therapy) have shown promise.^55^ Children and adolescents with greater sleep difficulties paired with additional problems (for example, substance use or depression symptoms) are generally more driven to change behavior given the downstream effects that insufficient sleep has on other aspects of their lives.^51^ A ‘one-size-fits-all’ approach is unlikely to be successful and individual sessions are better to enhance success.^51^ Tailored interventions aimed at improving adolescent sleep have shown better results.^56^ The location/setting, content, intensity, duration and the individual that delivers the program are all important features that need to be considered and that can predict the success of a sleep improvement program.^51, 55^

Overall, improving sleep in the pediatric population is not an easy task (especially in teenagers), and interventions should be individualized to maximize success. Addressing the key barriers on a case-by-case basis is important and it is advisable that sleep experts with a background in psychology deliver such interventions,^57^ particularly if interventions are supported by robust theories of behavior modification and include targeting multiple lifestyle components known to impact sleep (for example, screen time before bedtime, caffeinated beverages, nicotine and alcohol use, mental stress, peer/social influences, perceptions/beliefs and so on). Further studies are definitively needed in this field of research to (i) assess the feasibility of increasing/improving sleep in children and adolescents; (ii) identify key elements of success; and (iii) determine if this leads to improved health outcomes, including glucose homeostasis indicators.

## Importance of taking sleep more seriously from a public health standpoint

Until recently, T2D was rarely diagnosed in the pediatric population as evidenced by its previously popular name of adult-onset diabetes. The pervasiveness of T2D in children and adolescents is increasing and this trend is not solely seen in America but worldwide.^58^ The rates of diagnoses of T2D in the pediatric population are certainly worrisome but also of concern are the anecdotal reports supporting the notion that early T2D diagnosis in children is a disease that progresses at alarming rates compared with the disease progression of the adult-onset version of T2D.^59, 60^ Although T2D diagnosis in children is quickly becoming one of the most important public health concerns, it is also apparent that children’s sleep duration, quality and sleep–wake time schedule is becoming part of the issue. Sleep has an important role in the primary and secondary prevention of numerous cardiovascular diseases and metabolic conditions, including T2D.^3, 4, 61, 62^ Although it is undeniable that T2D in the pediatric population is a complex and multifaceted disease, presently there are scarcely any viable long-term solutions for the growing rates of T2D in children and adolescents. A multidisciplinary health-care approach with regular follow-ups has been shown to be the most successful at improving glycemic control in adults with T2D.^63^ However, currently there is little evidence to support this approach in children and adolescents with or at risk for T2D. A multidisciplinary approach is contingent on many factors, such as resource availability, location, expertise and subsidized programs.^64^ This holistic approach is family and patient centered and generally includes a large team of dedicated individuals, including a primary care physician, an endocrinologist, a registered nurse, a nutritionist or dietician, an exercise physiologist, a social worker, a psychologist and diabetes educators.^64^ As sleep is such a vital component of overall health and well-being (and also interacts with other behaviors), the team should, as common practice for both preventative and therapeutic purposes, ask about children and adolescents’ sleep quality, sleep duration and sleep–wake time schedule. We previously published an example of simple and quick questions that can be used to assess sleep as a vital health indicator.^65^ After assessing sleep, clinicians should be recommending that children and adolescents improve their sleeping habits. Although it is no easy task to get children and adolescents to adhere to recommendations, there is no harm in recommending more sleep and solutions should be individualized to the family by addressing root causes of the problem and finding feasible solutions. Some sleep duration guidelines do exist to help evaluate and recommend proper sleep duration. For example, the National Sleep Foundation in the United States recommends that toddlers aged 1–2 years sleep between 11 and 14 h per night, preschoolers aged 3–5 years sleep between 10 and 13 h per night, children aged 6–13 years sleep between 9 and 11 h per night and adolescents aged 14–17 years sleep between 8 and 10 h per night to maximize overall health and well-being.^66^

In keeping with the holistic approach to health, researchers in Canada, with the help from national agencies, have developed and released the world’s first evidence-based integrated 24-h movement guidelines aimed at optimizing health benefits of children and adolescents aged 5–17 years.^67^ These integrated 24-h movement guidelines include recommendations for moderate-to-vigorous physical activity, light physical activity, sedentary behaviors and sleep. These 24-h movement guidelines are the first in the world to include sleep recommendations alongside other movement behaviors. The sleep duration recommendations are in line with the ones stipulated by the National Sleep Foundation. These new Canadian guidelines are a step in the right direction when it comes to repositioning efforts to include sleep as a modifiable risk factor and a vital health indicator that is equally important as physical activity for overall health and well-being. A holistic approach may be a more realistic way of tackling a problem as complex as T2D in children and adolescents as the reductionist approach of addressing only physical activity and nutrition has yielded no clear long-term benefits for the increasing rates of T2D diagnosis.

## Conclusion

In summary, notwithstanding the fairly limited evidence in this population, all sleep characteristics appear to provide insights and are worth measuring to gain a better understanding of the association between sleep and glucose homeostasis in children and adolescents. Likewise, observational evidence tends to suggest that short sleep duration and poor sleep quality are associated with insulin resistance in children and adolescents. Furthermore, sleep architecture (namely SWS and REM sleep) appears to have relevance when it comes to glucose homeostasis in the pediatric population. The association between sleep and T2D biomarkers in children is often mediated by excess adiposity but other covariates, not always taken into account, are also especially important to consider in this population (for example, biological maturation, physical activity level, screen time, dietary habits), and not controlling for these variables is certainly an important limitation. There is a need for more research in order to better characterize subgroups of children more likely to be affected by insufficient sleep in order to better inform treatment and prevention strategies of T2D. Additionally, the need to pilot test novel interventions aimed at improving children and adolescent sleep and test whether it improves outcome measures (for example, T2D biomarkers) is needed. Although there is a necessity for more studies examining the link between insufficient sleep and the risk for T2D in the pediatric population (especially longitudinal studies and experimental trials), it is important in the meantime to take a pragmatic approach and encouraging a good night’s sleep as an adjunct to other health-promotion measures. It has become apparent to researchers in this field that sleep is not a waste of time and it has never been more apparent that children too need their sleep to remain healthy.

## Figures and Tables

**Table 1:** Observational studies on the association between sleep and glucose homeostasis in children and adolescents

*Reference*	*Study design*	*Age (years)*	n	*Sleep assessment*	*Outcome*	*Covariates*	*Main findings*
Androutsos *et al.*^27^	Cross-sectional	9–13	2026	Parent reported	HOMA-IR	Age, sex, Tanner stage, WC, parental BMI, SES index and birth weight	A lifestyle characterized by short sleep duration (⩽8.35±0.73 h per day), more screen time (⩾3.61±1.68 h per day) and higher consumption of sugared-sweetened beverages (⩾222.96±222.81 g per day) was associated with increased HOMA-IR (*β*=0.043, *P*=0.04). Note that the association between sleep duration alone and HOMA-IR was not reported
Armitage *et al.*^15^	Cross-sectional	13–18	18	1 night of PSG and prior to PSG 5 nights of sleep diary and actigraphy	HOMA-IR and WBISI	Age, BMI and Tanner stage	WBISI was not significantly associated with sleep characteristics after controlling for Tanner stage as a covariate. Those with highest HOMA-IR (13.1±6.2; *n*=4) had a significantly higher proportion of NREM1 and lower NREM2–4 than those with moderate HOMA-IR (5.6±1.0; *n*=8) and low HOMA-IR (3.0±2.2; *n*=6). Sleep duration did not significantly differ by HOMA-IR category
Azadbakht *et al.*^17^	Cross-sectional	10–18	5528	Parent reported	Fasting glucose	Age, SES index, parents’ education, family history of chronic disease, sedentary lifestyle and BMI	No association was found between sleep duration and fasting glucose in both boys and girls
Berentzen *et al.*^18^	Cross-sectional	11–12	1481	Self-reported questionnaire	HbA1c	Child’s age at the completion of questionnaire and medical examination, height, Tanner stage, screen time, storage time for blood sample and maternal education	No associations were found between sleep quality or duration and HbA1c in both boys and girls
Cespedes *et al.*^25^	Longitudinal	6 months–7 years	652	Parent reported	HOMA-IR, fasting glucose and fasting insulin	Age, sex, maternal education, prepregnancy BMI, number of previous pregnancy, age at enrollment, ethnicity, SES, and BMI *z*-score	After adding BMI *z*-score to the model, the association between sleep curtailment score and HOMA-IR and insulin fell short of significance. No association was found between sleep and fasting glucose
De Bernardi Rodrigues *et al.*^28^	Cross-sectional	10–19	615 (subsample for ISI 81)	Self-reported questionnaire	Fasting glucose, fasting insulin, ISI (via hyperglycemic clamp) and HOMA-IR	Age and sex	In the subsample (*n*=81), youth with short sleep duration (<8 h per night) had a lower median (IQR) ISI (assessed by hyperglycemic clamp) than those who slept an adequate duration per night (⩾8 h per night) (*β*=−0.01, 95% CI=−0.01; −0.00, *P*=0.02). In the large sample, no significant association were found between sleep duration and fasting glucose, fasting insulin and HOMA-IR
Flint *et al.*^29^	Cross-sectional	3–18	39	1 night of PSG	Fasting glucose, fasting insulin, peak insulin, IGI, HOMA-IR and WBISI	Age, BMI *z*-score, OSAS and Tanner stage	Compared with children and adolescents with a sleep duration of >6 h, those with ⩽6 h (*n*=14) had significantly higher fasting insulin (25.7±12.6 μU ml^−1^ vs 16.0±11.4 μU ml^−1^, *P*=0.02), higher peak insulin (226±142.3 vs 113.6±93.5 μU ml^−1^, *P*=0.02), higher HOMA-IR (3.3±2.4 vs 5.5±2.9, *P*=0.01) and lower WBISI (2.2±1.1 vs 7.0±6.2, *P*=0.01). No significant differences were observed between the two sleep duration groups for fasting glucose, IGI and glucose level 2 h after OGTT. The %REM sleep was significantly lower for the short sleepers (13.5±5.8%) compared with the longer sleeper group (18.6±5.7%)
Hitze *et al.*^19^	Cross-sectional	6–19	250	Self-reported questionnaire (children <11 years were also helped by parents)	HOMA-IR, fasting glucose and fasting insulin	Age and WC *z*-score	In girls (*n*=122), sleep duration was negatively correlated with both fasting insulin and HOMA-IR (both, *r*=−0.20, *P*=0.05); however, after controlling for WC *z*-scores the relationship was no longer significant. In boys, no correlations were found between sleep duration and all of the outcome measurements
Hjorth *et al.*^26^	Cross-sectional and longitudinal	8–11	723 (subsample for longitudinal sleep data 486)	8 nights of actigraphy (waist), sleep log (both self-reported and parent reported) and CSHQ (parent reported)	HOMA-IR	Cross-sectional: age, sex, Tanner stage, sex–pubertal status interaction, MVPA, sedentary time, and total physical activity Longitudinal: age, sex, Tanner stage, sex–pubertal status interaction, MVPA, sedentary time, total physical activity, and changes in fat mass index	Cross-sectional data (*n*=719) revealed that sleep problems noted by parents in the CSHQ were positively associated with HOMA-IR (*β*=0.007, 95% CI=0.002; 0.013) Sleep duration (*n*=473) was negatively associated with HOMA-IR (*β*=−0.080, 95% CI=−0.174; 0.014). Longitudinal data (*n*=486) showed that changes in sleep duration were negatively associated with changes in HOMA-IR (*β*=−0.18, 95% CI=−0.36; 0.01)
Javaheri *et al.*^30^	Cross-sectional	15.7±2.1	471	5–7 nights of actigraphy (wrist)	HOMA-IR and fasting insulin	Age, sex, ethnicity, preterm status, MVPA and WC	Adolescents who slept 10.5 h had the highest predicted HOMA-IR (2.33; 95% CI=1.97; 2.76) while not statistically significant HOMA-IR levels were approximately 30% lower in adolescents who slept 7.75 h and 22% lower in adolescents who slept 5 h (1.78; 95% CI=1.67; 1.91 and 1.93; 95% CI=1.62; 2.30, respectively)
Koren *et al.*^31^	Cross-sectional	8–17	62	1 night of PSG	OGTT, HbA1c, FSIGT, insulin levels, glucose levels, HOMA-IR,WBISI, IGI and AIRg	Age, sex, Tanner stage, OSAS and BMI *z*-score (degree of obesity)	In adolescents with obesity, data displayed a U-shaped association between sleep duration, fasting glucose (*R*^2^ quadratic=0.201, *P*=0.002), 2-h glucose (*R*^2^ quadratic=0.442, *P*<0.001) and HbA1c (*R*^2^ quadratic=0.200, *P*=0.002). NREM3 sleep duration was a strong predictor of insulin level as indicated by IGI (*R*^2^ quadratic=0.161, *P*=0.002) and AIRg (*R*^2^ quadratic=0.383, *P*<0.001). A positive correlation was shown between NREM3% of total sleep and 2-h insulin plasma level (*r*=0.348, *P*<0.01). A negative correlation was found not only between NREM2 duration and fasting insulin level (*r*=−0.267, *P*<0.05) and HOMA-IR (*r*=−0.282, *P* <0.05) but also between NREM2% of total sleep and 2-h insulin plasma level (*r*=−0.280, *P*<0.05)
Lee and Park^16^	Cross-sectional	12–18	1187	Self-reported questionnaire	Fasting glucose	Age, sex, SES, caloric intake and physical activity	No significant association was found between sleep duration and fasting glucose
Matthews *et al.*^32^	Cross-sectional	14–19	245	7 nights of actigraphy (wrist) and sleep diary	HOMA-IR, fasting glucose and insulin	Age, sex, ethnicity, WC *z*-score and BMI residual	The HOMA-IR was negatively associated with weekday sleep duration measured by both actigraphy and sleep diary (*β*=−0.211, 95% CI −0.314; −0.107, *P*<0.001, *β*=−0.147, 95% CI −0.249; −0.046, *P*=0.005, respectively) and total sleep duration measured by both actigraphy and sleep diary (*β*=−0.202, 95% CI −0.307; −0.096, *P*<0.001, *β*=−0.145, 95% CI −0.248; −0.043, *P*=0.006, respectively) However, HOMA-IR was not associated with weekend sleep duration (*β*=−0.054, 95% CI −0.158; 0.049, *P*=0.306). No associations were found between sleep duration and fasting glucose. However, sleep fragmentation was positively associated with fasting glucose (*β*=0.140 mg dl^−1^, *P*=0.035) but not associated with HOMA-IR
Navarro-Solera *et al.*^20^	Cross-sectional	7–16	90	Self-reported questionnaire	Fasting glucose, fasting insulin and HOMA-IR	Age, sex, BMI, physical activity and KIDMED index	No significant association was found between sleep duration and HOMA-IR, fasting glucose or insulin
Prats-Puig *et al.*^33^	Cross-sectional	5–9	297	Self-reported questionnaire with parental help	HOMA-IR	Age, sex, nutrition, physical activity and family history of obesity	No association was found between sleep duration and HOMA-IR in the overall sample. Sleep duration was negatively associated with HOMA-IR in children of a specific phenotype (that is, NRXN3 rs10146997 G) (*β*=−0.171; 95% CI=−0.276; −0.066)
Rey-López *et al.*^21^	Cross-sectional	12–17	699	Self-reported questionnaire	HOMA-IR	Age, sex, SES and MVPA	No association was found between sleep duration and HOMA-IR
Spruyt *et al.*^22^	Cross-sectional	4–10	107	7 nights of actigraphy (wrist)	Glucose and insulin	Age, sex, ethnicity and BMI *z*-score	No associations were found between sleep duration and glucose or insulin concentrations
Sung *et al.*^23^	Cross-sectional	10–16	133	7 nights of actigraphy (wrist) accompanied by sleep log and questionnaires (parent reported and self-reported)	Fasting glucose and HOMA-IR	Age, sex, ethnicity, SES, BMI *z*-score and OSAS	No associations were found between sleep duration and HOMA-IR or fasting glucose
Tian *et al.*^34^	Cross-sectional	3–6	1236	Parent reported	Fasting glucose	Age, sex, birth weight, gestational age, SBP, parent’s education, BMI *z*-score, WC, diseases in the past month, breastfeeding at 6 months, diet and nutrition, screen time and physical activity	A negative association between sleep duration and fasting glucose was found (*β*=−0.043, s.e.=0.021, *P*=0.04). An increased risk of hyperglycemia (⩾100 mg dl^−1^) for those sleeping ⩽8 h compared with those sleeping 9–10 h was observed (OR=1.64, 95% CI=1.09; 2.46). When stratified by weight status, the association was only present in obese children (OR 2.15, 95% CI=1.20; 3.84)
Turel *et al.*^24^	Cross-sectional	10–17	94	Fitbit activity watch accompanied by a sleep log	Fasting glucose, fasting insulin and HOMA-IR	Age, sex, SES, BMI *z*-score, WC and medications	No association was found between sleep duration and HOMA-IR, fasting glucose or fasting insulin
Zhu *et al.*^35^	Cross-sectional	13.1±3.3	118	1 night of PSG	OGTT, insulin, glucose, IS_OGTT_ and ISSI-2	Age, sex, BMI *z*-score, Tanner stage and OSAS	The 2-h glucose was negatively associated with total sleep time and sleep efficiency (*β*=−9.96 × 10 ^−4^, s.e.=3.23 × 10^−4^, *P*<0.001 and *β*=−0.005, s.e.=0.002, *P*=0.011, respectively). A positive association was observed between IS_OGTT_ and sleep efficiency and NREM3% of total sleep time (*β*=0.013, s.e.=0.005, *P*=0.016, and *β*=0.024, s.e.=0.009, *P*=0.012, respectively). IS_OGTT_ was also negatively associated with NREM1% of total sleep time (*β*=−0.058, s.e.=0.025, *P*=0.021) ISSI-2 was positively associated with both total sleep time and sleep efficiency (*β*=0.002, s.e.=0.001, *P*=0.008, and *β*=0.010, s.e.=0.004, *P*=0.014, respectively)

Abbreviations: AIRg, acute insulin response to glucose; BMI, body mass index; CI, confidence interval; CSHQ, children’s sleep habits questionnaire; FSIGT, frequently sampled intravenous glucose tolerance test; HbA1c, glycated hemoglobin; HOMA-IR, homeostasis model assessment of insulin resistance; IGI, insulinogenic index; IQR, interquartile range; ISI, insulin sensitivity index; IS_OGTT_, insulin sensitivity index for oral glucose tolerance test; ISSI-2, insulin secretion sensitivity index 2; KIDMED index, Mediterranean Diet Quality Index for Children and Adolescents; MVPA, moderate-to-vigorous physical activity; NREM, non-rapid eye movement; OGTT, oral glucose tolerance test; OR, odds ratio; OSAS, obstructive sleep apnea syndrome; PSG, polysomnography; REM, rapid eye movement sleep; SBP, systolic blood pressure; SES, socioeconomic status; WBISI, whole-body insulin sensitivity index; WC, waist circumference. Note: Main findings from analyses of sleep duration are treated as categorical variables and presented as mean±s.d. unless stated otherwise. Main findings represent the most adjusted models unless stated otherwise.

**Table 2:** Experimental studies on the association between sleep and glucose homeostasis in children and adolescents

*Reference*	*Study design*	*Age (years)*	n	*Sleep assessment*	*Experimental groups*	*Outcomes*	*Covariates*	*Main findings*
Klingenberg *et al.*^36^	Randomized crossover	15–19	21	3 nights of PSG (2 conditions)	Two conditions: short sleep (4 h per night) and long sleep (9 h per night)	HOMA-IR, Matsuda index, glucose, insulin and C-peptide	Age, PSQI, diet and physical activity	This study conducted with male adolescents of normal weight showed that during the long sleep condition the adolescents had significantly lower fasting insulin levels (15.3±3.1, 95% CI −14.0; −4.2 vs 24.4±4.8, *P*=0.001), lower fasting C-peptide (414.8±30.4, 95% CI −146.1; −53.2, vs 514.4±37.0, *P* <0.001), less area under and over the curve for C-peptide (1758.3±96.3, 95% CI −354.8; −38.4 vs 1954.9±94.3, *P*=0.018) and lower HOMA-IR (0.46±0.11, 95% CI−0.50; −0.11, vs 0.76±0.18, *P*=0.002) than the short sleep group. The long sleep group also had a higher Matsuda index (49.5±7.0, 95% CI 2.9; 19.9, vs 38.8±6.0, *P*=0.007) than the short sleep group. There were no significant differences found between the two sleep groups for fasting blood glucose and for areas under and over the curves for glucose and insulin
Shaw *et al.*^37^	Randomized crossover	11–14	14	2 nights of PSG (2 conditions)	Two conditions: with and without slow wave sleep disruption	Fasting insulin, fasting glucose, C-peptide and HOMA-IR	Age, sex, Tanner stage, ethnicity and BMI percentile	No associations were found between HOMA-IR, fasting insulin, fasting glucose and C-peptide and minutes spent in slow wave sleep (*β*=0.04, *P*=0.1) or between the percentage of change in slow wave sleep and percentage of change in HOMA-IR (*β*=0.03, *P*=0.6)

Abbreviations: BMI, body mass index; CI, confidence interval; HOMA-IR, homeostasis model assessment of insulin resistance; PSG, polysomnography; PSQI, Pittsburgh Sleep Quality Index. Note: Main findings are presented as mean±s.d. Main findings represent the most adjusted models.
